# The Comprehensive “Omics” Approach from Metabolomics to Advanced Omics for Development of Immune Checkpoint Inhibitors: Potential Strategies for Next Generation of Cancer Immunotherapy

**DOI:** 10.3390/ijms22136932

**Published:** 2021-06-28

**Authors:** Sang Jun Yoon, Chae Bin Lee, Soon Uk Chae, Seong Jun Jo, Soo Kyung Bae

**Affiliations:** College of Pharmacy and Integrated Research Institute of Pharmaceutical Sciences, The Catholic University of Korea, 43 Jibong-ro, Wonmi-gu, Bucheon 14662, Korea; ysj6416@catholic.ac.kr (S.J.Y.); aribri727@catholic.ac.kr (C.B.L.); zldtnseo@catholic.ac.kr (S.U.C.); sungjun6734@catholic.ac.kr (S.J.J.)

**Keywords:** cancer immunotherapy, immune checkpoint inhibitor, metabolomics, omics, microbiome, immune-related adverse events

## Abstract

In the past decade, immunotherapies have been emerging as an effective way to treat cancer. Among several categories of immunotherapies, immune checkpoint inhibitors (ICIs) are the most well-known and widely used options for cancer treatment. Although several studies continue, this treatment option has yet to be developed into a precise application in the clinical setting. Recently, omics as a high-throughput technique for understanding the genome, transcriptome, proteome, and metabolome has revolutionized medical research and led to integrative interpretation to advance our understanding of biological systems. Advanced omics techniques, such as multi-omics, single-cell omics, and typical omics approaches, have been adopted to investigate various cancer immunotherapies. In this review, we highlight metabolomic studies regarding the development of ICIs involved in the discovery of targets or mechanisms of action and assessment of clinical outcomes, including drug response and resistance and propose biomarkers. Furthermore, we also discuss the genomics, proteomics, and advanced omics studies providing insights and comprehensive or novel approaches for ICI development. The overview of ICI studies suggests potential strategies for the development of other cancer immunotherapies using omics techniques in future studies.

## 1. Introduction

The International Agency for Research on Cancer (IARC), as a part of the World Health Organization (WHO), announced that cancer is the second highest cause of death and is associated with approximately 10 million deaths per year based on the WHO cancer mortality database. To alleviate the mortality of cancer, various therapeutic agents have been developed, ranging from chemotherapy to immunotherapy [1]. Although chemotherapy, as the first generation of cancer therapy, has been the prevalent option for cancer treatment with radiotherapy and surgery in the past decades, the development of tyrosine kinase inhibitors and monoclonal antibodies for cancer, second-generation therapeutic agents, have improved the efficiency of therapies derived by that high-specificity and broad therapeutic window [2]. In recent decades, among several therapeutic approaches, cancer immunotherapy is considered the third generation of cancer therapy, which can overcome the limitations of previous approaches. Cancer immunotherapy, sometimes called immune-oncology, directly or indirectly stimulates the host immune system to control or eliminate cancer [3]. Various strategies have been developed to evoke patients’ own tumor immunity, such as immune checkpoint inhibitors (ICIs), immune cell therapy, and anti-cancer vaccines, but ICIs are the most well-studied category of immunotherapies.

‘Omics’ is a high-throughput technique for the investigation of biological systems, including diverse molecular layers (e.g., genes, proteins, and metabolites), and supports a large number of datasets, such as those of the genome, transcriptome, or metabolome representing biological dynamics [4]. To date, omics-based systems biology has significantly contributed to advances in ontology while considering its numerous applications for cancer research, such as biomarker discovery, therapeutic target suggestions, and prognosis and diagnosis assessment. Likewise, these omics-based platforms have developed immunotherapy known as third-generation cancer therapy. The advantages of omics techniques include (1) supporting reliable high-throughput datasets across several molecular layers, which enable integration for comprehensive biological interpretations, (2) simultaneous achievement of qualification and quantification for many molecules for effective target screening, (3) high availability and applicability using reasonable costs and labor derived from the development of mass spectrometry and sequencing technology. Several studies regarding cancer immunotherapy, especially ICIs, have applied omics and highlighted several outcomes, such as extensive targets for drug discovery and development, determination of mechanisms of action, pre- or post-treatment biomarkers of clinical outcomes, and assessment of immune-related adverse events [5].

In this review, we discuss the recent application of omics techniques in the study of immune checkpoint inhibitors and extend the knowledge of omics-based approaches to advance our understanding of cancer immunotherapy for future studies.

## 2. Emerging Application of Immunotherapy for Cancer

Since the concept that the immune system can recognize and prevent carcinogenesis at early stages was introduced in 1909 by Paul Erlich, it was incorporated into the ‘cancer immunosurveillance’ theory by Burnet and Thomas in the middle of the 19th century [6]. The controversial theory was based on several studies conducted in various cancer models, providing evidence either confirming or opposing the theory. Following continuous studies, the theory was developed into the concept of ‘cancer immune-editing,’ in that the immune system demonstrates not only host-protection but also tumor-sculpting effects on cancer development [7]. Diverse components of the immune system protect the host against nascent cancer development or improve tumor escape or both by cancer immune-editing [8]. The process is divided into three phases, including elimination, equilibrium, and escape [9]. In the elimination phase, the immune system is able to destroy the tumor through the action of NK, CD4+, and CD8+ cells. However, equilibrium between immune system components and tumor cells leads to the failure of tumor suppression at the second stage. Finally, the tumor acquires immune evasion abilities and becomes detectable in the escape phase.

Recently, the tumor microenvironment (TME), which is the surrounding environment interacting with tumors, has been considered as an emerging field in cancer study and re-establishes drug efficacy and therapeutic strategies in cancer immunotherapy [10]. TME includes various components, such as blood vessels, immune cells, fibroblastic cells, and extracellular matrix [11]. The tumor and TME interact closely, resulting in increased tumor heterogeneity [12]. An improved understanding of the TME demonstrated that immune cell infiltration has a high correlation with anti-cancer immune responses, and it led to the creation of the ‘immune contexture,’ involving organization, composition, and density of immune cell infiltrate [13]. Following the specific categories of the concept, several therapeutic trials were conducted to suggest precision medicine [14]. These findings demonstrated that tumor interaction with immune systems and the TME could serve as a crucial target for cancer immunotherapy.

After the first attempt at using the immune system for cancer therapy in the late 19th century, immune-oncology or immunotherapy has been developed continuously and has become an anticipated field, as evidenced by the Nobel prize for physiology or medicine being awarded T-cell to Drs. Allison and Honjo in 2018 for the discovery of T-cell immune checkpoints, such as CTLA-4 and PD-1 [15,16]. Cancer immunotherapy, regarded as a third-generation cancer therapy, modifies the patient’s own immune system to control or eliminate cancer [17]. To date, the typical types of cancer treatment are chemotherapy, radiation therapy, surgery, and a combination of those options. However, chemotherapy, known as a first-generation anti-cancer treatment, displays many side effects, including fatigue, nausea, vomiting, hair loss, and pain, due to the fact that the treatment cannot distinguish between tumor and host cells [18]. To overcome the limitation of chemotherapy, a second-generation targeted therapy was developed to improve specificity to block cancer growth, progression, and metastasis, followed by an extension of the therapeutic window [19]. Although this strategy increased specificity, universal application was difficult for various types of cancer, and drug resistance occasionally occurs [20]. Meanwhile, cancer immunotherapy activates patients’ own immune systems, which are suppressed by cancer immune-editing, to destroy tumor cells. Thus, it has been used in diverse cancer types without additional gene manipulation in that immune responses are under strict regulation by immune checkpoints, and it increases the quality of treatment in accordance with the extension of survival rate and minimized side effects [21].

Although traditional immune therapy, including tumor vaccines, cytokine therapy, and adaptive cell transfer (ACT), has been used in specific cancers, insufficient effects and severe toxicities of this approach escalated the need for novel cancer therapeutics [22,23]. Following general classification, by which cancer immunotherapies are divided into “inactive (or passive)” and “active” according to their abilities to activate the immune system against tumor cells, tumor-targeting monoclonal antibodies and adoptively transferred T-cells are included in inactive immunotherapy, while anti-cancer vaccines and checkpoint inhibitors are considered as active immunotherapy [24]. Beginning with FDA approval for first-generation cancer immunotherapy, including sipuleucel-T (Provenge^®^; Dendreon) for prostatic cancer and ipilimumab (Yervoy^®^; Bristol-Meyers Squibb) for melanoma, immunotherapy has become the fastest-expanding area in cancer therapeutics [25,26]. Further, a blockade of immune checkpoints has been developed through second-generation immunotherapy, including PD-1 and PD-L1 antibodies following ipilimumab (CTLA-4 inhibitor) [27]. As of May 2021, eight immune checkpoint inhibitors have been approved by the FDA (Table 1).

Despite the potential for clinical benefits, low response rates and several resistance mechanisms have not yet been resolved. A critical limitation of immunotherapies is immune-related adverse events (irAEs), which are characterized by host immune activation against healthy cells [28]. The mortality rate due to severe myocarditis, an irAE, was 46% in immunotherapy-treated patients who received ICIs [29]. Moreover, PD-1 and CTLA-4 inhibitors result in the doubling of serious irAEs, although the survival rate of patients increases [30]. Fortunately, several markers for irAEs have been suggested, but the validation of these markers should be performed [31,32]. The insufficient characteristic of second-generation treatments led to the emergence of new generations with various novel therapeutic modalities based on novel strategies, which have been designed to elicit immune responses against tumors [27,33].

Two main strategies are the activation of co-stimulatory receptors and inhibition of immunosuppressive ligands or metabolism [33]. Agonistic monoclonal antibodies targeting tumor necrosis factor receptors, such as OX40, GITR, and CD137 expressed on several immune cells, provoke the extension of CD8+ T-cell survival, increasing tumor-specific T-cell responses, the upregulation of NK cells, and the regulation of regulatory T-cells [34,35,36]. Conversely, several studies have been reported regarding the inhibition of immunosuppressive targets, such as VISTA (v-domain Ig suppressor of T-cell activation), a ligand, and IDO1 (indoleamine 2,3-dioxygenase-1), an enzyme catalyzing the kynurenine pathway as a rate-limiting step for enhancing anti-tumor T-cell responses and inhibition of immune responses by depletion of tryptophan [37,38]. Based on the robust concept of immunotherapy established through past decades, the latest therapeutic trends include improvement of efficacy, management of response and toxicity, and extension of applicable targets [39]. To improve the efficacy using developed immunotherapies, several pharmaceutical and combinational strategies have been applied. Nanotechnology has become an effective option for eliciting immune responses by expanding the therapeutic window and enhancing vaccination or endogenous immune responses [40]. Similarly, PEGlyation, conjugation of polyethylene glycol (PEG) polymer to proteins, has been utilized to increase the half-life of cytokines in vivo [22]. Another method used to enhance efficacy is immunotherapy combined with typical cancer therapy, such as radiotherapy or immunomodulatory drugs [41,42]. Recent studies focus on biomarkers for immune response, including toxicity, as well as the establishment of guidelines for irAE [43,44]. Thus, various studies are continuously performed to discover biomarkers not only for immune responses but also for novel therapeutic targets using omics, which are most widely used in systems biology. Furthermore, many potential drugs are under clinical trials registered with ClinicalTrials.gov (https://clinicaltrials.gov/) (assessed on 21 May 2021) (Table 2).

## 3. Development of Omics Workflows for Cancer Immunotherapy Including ICIs

**Figure 1:**
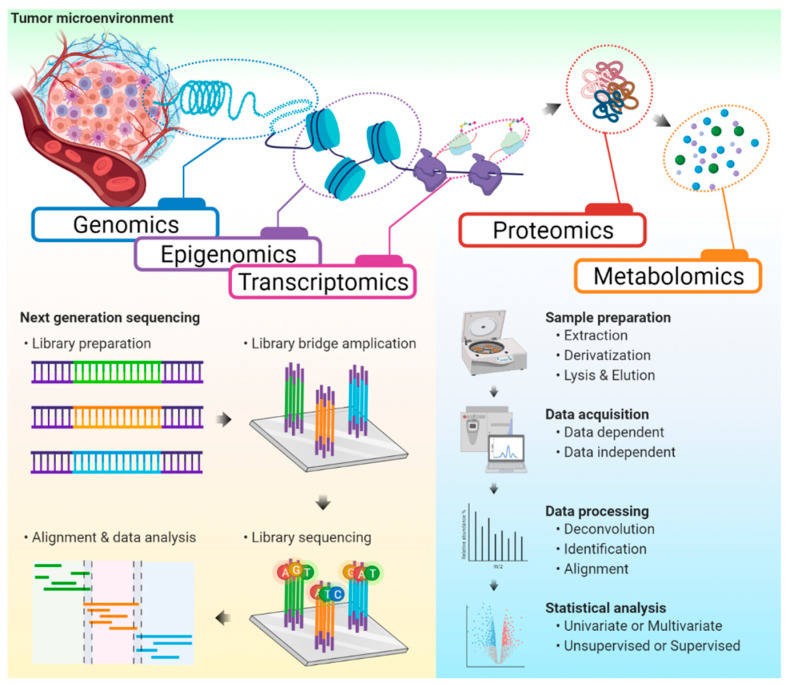
The workflow of omics platforms. Various omics approaches applied for the study of cancer immunotherapy were demonstrated with general workflows of next-generation sequencing and mass spectrometry-based omics.

Several omics platforms that provide high-throughput information for biological understanding have been applied to study cancer immunotherapy (Figure 1). In this section, we will deliberate about how these approaches have contributed to the development of ICIs and how they have given insights for future studies involving cancer immunotherapy.

### 3.1. Development of Omics Technologies

Since the human mitochondrial genome was identified about 40 years ago, genetics has been extensively applied to determine the biochemical function of the genome to further study cancer based on human genome sequences [45]. Following the enhancement of demands for high-throughput data, researchers began to realize the importance of the ‘systems biology’ approach for systemic biological interpretation and accelerated the development of several platforms. ‘Omics,’ as a technique to analyze large amounts of data comprising an entire set of analytes, includes various branches, such as genomics, transcriptomics, proteomics, metabolomics (or metabonomics), and lipidomics, and it aims to provide molecular profiles for understanding diverse biological dynamics [46]. In oncology, omics has been frequently used to demonstrate hallmarks, discover biomarker candidates for diagnosis or prognosis, determine target pathways, indicate mechanisms of drug response, and predict or assess toxicity, providing an effective method to develop therapeutic intervention [47,48]. Multiple analytical platforms that support high-throughput data derived from biological samples have been developed over the years. Microarray and next-generation sequencing (NGS), including whole-genome sequencing (WGS) or transcriptome sequencing, which overcame disadvantages associated with the Sanger sequencing method, have been broadly used for DNA and RNA samples. Exome sequencing and epigenome sequencing were additionally developed to improve the quality of target gene analysis. Recently, genotyping using polymerase chain reaction (PCR) or clustered regularly interspaced short palindromic repeats (CRISPR) is also applied for the development of cancer therapies. Moreover, the development of mass spectrometry technology and the method of data acquisition facilitates the broad application of proteomics, metabolomics, and lipidomics. Simultaneously, diverse tools supporting data pre-processing of raw data (e.g., data collection and gap-filling) and post-processing workflow for interpretation (e.g., biomarker analysis, network analysis, and pathway enrichment analysis) have been established [49,50,51,52]. Extensive data from several biochemical analytes are recruited to establish databases or in silico libraries (e.g., TCGA, TCPA, LipidBlast, and FiehnLib) by several societies or analytical teams for future studies [53,54,55,56].

### 3.2. Advantages of Omics-Based Systems Biology for Oncology

Omics-based systems biological approaches have several advantages in cancer study. First, omics supports reliable high-throughput datasets over several molecular layers (e.g., genome, transcriptome, proteome, and metabolome), which enable the integration for comprehensive biological interpretations, in that it suggests alteration of metabolism, tumor microenvironment (TME), and provides clues regarding tumor mechanisms. For example, diffuse gastric cancers were studied by integration of proteomic and genomic analyses and indicated an association between mRNA-protein abundance and patient survival [57]. Meanwhile, Kang et al. suggested the crucial roles of extracellular cystine in influencing the mechanism of ferroptosis in non-small cell lung cancer through stable isotope labeling-based metabolomics [58]. Second, omics provides the simultaneous achievement of qualitative and quantitative results for many molecules for effective therapeutic target screening, such as biomarkers or pathways associated with pre- or post-treatment clinical outcomes [59]. The clinical outcomes promote precision medicine through positive response or prevention of unintended negative effects, such as including toxicity and drug resistance [60,61]. Third, omics has high availability and applicability using reasonable cost and labor derived from the continuous development of mass spectrometry and sequencing technologies.

### 3.3. Recent Trends and Advanced Omics Platforms in Cancer Study

Several methodologies have been established for a systems biological approach in oncology to overcome the limitations of typical omics study. Multi-omics, also called pan-omics, is a biological analysis for the simultaneous integrated interpretation of multiple omics data sets [62]. Although comprehensive interpretation based on so-called multi-layer omics, which deduces results according to post-analysis integration, induces a better understanding of biological phenomena than typical single omics, it is complicated and usually conducted by knowledge-based interpretation, including the possibility of bias. Conversely, multi-omics is performed by specialized tools used for combining different omics data sets before further analyses [63]. Therefore, it elucidates potential causative alterations, which may become promising targets for cancer therapy, rather than reactive processes derived from analysis of one omics data set. Despite its powerful support for integrational interpretation, multi-omics need a logical strategy to link each data sets based on the evidence for causation, presenting functional associations between diverse molecular levels and preventing coincidental correlation [64]. Meanwhile, mass spectrometry imaging focuses on visualizing the spatial distribution of molecular targets to overcome the limitations of typical sample preparation methods, which pool all molecules into the same solvent [65]. Therefore, it is especially useful to gain this biochemical information [66]. Regarding mechanistic studies, stable isotope tracing is the most-developed technique to demonstrate the flux of target metabolism [67]. Furthermore, the combination of stable isotope tracing with other techniques, such as metabolomics or MSI, provides novel insights for understanding cancer metabolism and drug development [68,69]. Recently, genome engineering using the CRISPR-Cas9 system, which is the RNA-guided Cas9 nuclease from the microbial clustered regularly interspaced short palindromic repeats, has emerged [70]. Owing to precise genome editing by this technology, genome-wide CRISPR-Cas9 knockout screens were developed to determine the correlation between genotype and phenotype, connected with the development of cancer therapy [71].

### 3.4. Drug Development for ICIs Based on a Metabolomics Approach

According to the central dogma, genetic information in DNA has passed into protein through RNA, and these proteins regulate the intermediates (e.g., polar metabolites and lipids) associated with metabolic pathways, which can affect phenotypes [72]. In tumorous circumstances, the flow is distorted by several reasons and results in the expression of various cancer hallmarks, demonstrating cellular metabolism that has deviated from normal conditions. Thus, identification or quantification of these intermediates is essential to provide an understanding of metabolic reprogramming to the alteration of phenotypes by cancer. Metabolomics, an omics field, focuses on the study of small molecules, is a high-throughput technique for the parallel assessment of large-scale metabolites. Owing to the remarkable development of instrumental technologies and bioinformatics, the proportion of metabolomics contributing to the systems-level understanding of diseases has increased in the past decades. As well as high accessibility, synergistic effects derived from the inherent importance of the metabolome and flexible applicability based on large-scale datasets lead to frequent use for the identification and validation of metabolic profiles, simultaneous large-scale quantification, and functional analysis of the metabolome [73]. In addition, recent advances regarding metabolome information-based fluxomics and stable isotope tracing enable a greater understanding of disease mechanisms, including cancers [68,74].

Although various methods have been developed and selected by sample matrix, chromatography-coupled mass spectrometry (MS) is the most powerful and broadly used technique for metabolomics. Three important considerations for LC/MS- or GC/MS-based metabolomics are sample preparation, chromatography conditions, and MS compartment. The extraction method and solvent are selected based on the characteristic of interest, and the ultimate goal of optimization is a reproducible technique to extract several metabolites [75,76]. Meanwhile, chromatography conditions determine the separation of numerous metabolites through interaction with stationary and mobile phases and detect metabolites using MS in a time-dependent manner [77,78]. MS coupled with chromatography is an important technique and is most often used in metabolomics. For detection by a mass spectrometer, molecules must be ionized in specific ways, of which ESI and electron impact are frequent methods in LC and GC, respectively.

Recently, metabolomic approaches have been used in several developments related to immunotherapy and highlight changes in downstream molecules (e.g., amino acids, nucleic acids, and lipids) as a result of aberrant upstream signals, which play important roles for metabolic pathways directly related to the expression of crucial phenotypes. Based on this advantage, metabolomics has been applied to investigate novel therapeutic targets for cancer immunotherapy and identify promising metabolic biomarkers for the assessment of post-treatment outcomes or pre-treatment predictors. Further, metabolomics methods are continuously developed and optimized for the study of ICIs [79,80]. Furthermore, this technique has expanded the range of biological interpretation through comprehensive interpretation with other omics approaches (e.g., genomics, proteomics, transcriptomics, and metagenomics) and suggested novel strategies for the development of immunotherapies, such as the association between microbiota metabolites with ICI efficacy and the discovery of immune system-related metabolic pathways (e.g., kynurenine pathway). Herein, we summarized previous studies involving ICIs or focused on the development of ICIs among several studies regarding cancer immunotherapy (Table 3).

#### 3.4.1. Target Discovery

During the development of novel therapeutic approaches for specific diseases, including cancer, the top priority is the identification of suitable and effective targets for therapy. One of the key considerations for effective ICI is the dysregulated metabolism of T-cell-mediated tumor microenvironments, and it provides novel therapeutic targets based on the improved understanding of the interplay between functional states of T-cells and immune metabolism. For example, Palaskas et al. conducted a mass spectrometry-based metabolomics study in vitro to investigate metabolic alterations affected by PD-1 signaling. They demonstrated that PD-1 signaling for non-adherent primary human T-cells prevented de novo nucleoside phosphate synthesis accompanied by decreased mTORC1 signaling, while exogenous purines and pyrimidines failed to rescue the proliferation of PD-L1-treated cells [81]. Liu et al. reported an association between lipid metabolism and T-cell senescence, suggesting that reprogrammed lipid metabolism was triggered by the upregulation of PLA2G4A in cancer cells and regulatory T-cells. Using melanoma and breast cancer in vivo models, they demonstrated enhanced therapeutic efficacy following inhibition of PLA2G4A [82]. Moreover, another study identified the negative correlation between response to tumor-infiltrating lymphocyte (TIL) therapy and upregulation of Sirt2 in human TILs. This study indicated that Sirt2-deficient T-cells increased antitumor activity resulting from upregulated oxidative phosphorylation and glycolysis, following the enhancement of effector functions and proliferation [83]. A remarkable finding regarding immunotherapy indicated that tryptophan metabolites related to the kynurenine pathway and IDO-1 activity were potential targets of novel immunotherapy. Heng et al. performed kynurenine pathway profiling using large-scale clinical samples from patients with several types of breast cancer. They revealed potent immunosuppression by increased anthranilic acid and 2-hydroxylanthranilic acid derived from downregulation of kynurenine monooxygenase and kynureninase in triple-negative and HER2-enriched breast cancer subtypes [105]. Furthermore, many recent studies have reported an association between the immune checkpoint and tryptophan metabolism related to IDO-1/TDO. Some studies have suggested IDO-1 as an indicator or potential target for combination therapy. Kesarwani et al. recommended a combination of IDO-1 inhibitors with radiotherapy for increased therapeutic efficacy by preventing RC-induced immunosuppression [84]. Kocher et al. identified alterations in 67 metabolites in NSCLC patients receiving ICI treatment compared with healthy controls using LC-MS/MS, indicating dysregulation of IDO activity in patients. Based on the results, they suggested tryptophan as a promising biomarker for ICIs [85]. Besides, the discoveries of compensatory or combinational targets for ICIs have been continued. Sadik et al. identified that the activation of aryl hydrocarbon receptor (AHR) reduced anti-tumor immunity, and interleukin-4-induced-1 (IL4I1) was associated with AHR activity more than IDO-1 or TDO2 [86]. Other studies under anti-PD-1 conditions were used to assess the response by drug treatment and investigate novel targets. For example, PRMT5 inhibition demonstrated synergistic mechanisms enhancing anti-tumor immunity and alleviated resistance to ICIs [87]. A lipidomics approach demonstrated that the upregulation of sphingomyelin phosphodiesterase 3 by sphingomyelinase 2 (nSMase2) is a potential strategy to overcome resistance against PD-1 inhibitors according to increased PD-1 inhibitor efficacy following over-expression of wild-type nSMase2 in melanoma [88].

#### 3.4.2. Discovery of Biomarkers and Efficacy Evaluation

Although there is no qualified biomarker related to cancer immunotherapy by U.S. FDA until now, the discovery of biomarkers to evaluate outcomes post-treatment and to predict responses pre-treatment is important for therapy development, as well as finding novel targets. A study using metabolomics for PDAC under anti-PD-1 and anti-CTLA-4 conditions demonstrated increased sensitivity of ICIs by IL17 inhibitor resulting in prevention of cytotoxic CD8 T-cell exclusion from tumors and suggested that tumor lactate may serve as a promising early biomarker for efficacy of IL17/PD-1 combination [93]. Given that the immune mechanism is related to the kynurenine pathway and kynurenine to tryptophan ratio, it contributed to the development of a marker of tumor aggressiveness and metabolic profiling alteration in response to treatment with PD-1 inhibitors (e.g., nivolumab and pembrolizumab). Based on the association between increased serum kynurenine/tryptophan ratio and worse overall survival, the combination of IDO/TDO inhibitors and PD-1 inhibitors has been advocated [94,106]. Additionally, a comprehensive evaluation of metabolites in serum from ICI-treated patients demonstrated that very-long-chain fatty acid (VLCFA) containing lipids predicted efficacy and therapy response [95]. Moreover, biomarkers can be used to evaluate drug efficacy. Karayama et al. analyzed plasma from 19 ICI-treated patients with NSCLC and identified tryptophan metabolites. Based on the interpretation of the metabolite intensities with drug response and survival rate, they suggested tryptophan metabolites as potential predictors of ICI efficacy [96]. Another study also suggested the novel NMR-based metabolomics approach, providing metabolomic serum fingerprints for the predictive assessment of ICI efficacy, and it showed more than 80% accuracy in 50 patients with NSCLC receiving nivolumab and pembrolizumab treatments [97]. Regarding biomarker discovery and efficacy evaluation, metabolomics is most frequently used to study the microbiome under ICI conditions. Stool is a commonly used sample for microbiome studies; thus, several studies have optimized and applied unbiased metabolomic profiling methods for fecal samples [98]. Some studies applied GC-MS/SPME-based metabolomics for the detection of volatile organic compounds (VOCs) and NMR-based metabolomics for non-VOCs to investigate the gut metabolome involved in nivolumab treatment for NSCLC. These studies introduced metabolomic approaches and their network analysis as promising strategies for the management of cancer patients and prediction of good responders using microbiota-linked indicators [99,100]. In addition, another study proposed integrated parameters, including gut metabolites and immunological molecules from serum and stool for identification of nivolumab responders before treatment [101].

### 3.5. Metabolomics and Metagenomics for Identifying Interactions between the Microbiome and ICIs

Although metabolomics, including lipidomics, play key roles to evaluate the efficacy of therapies and discover predictive biomarkers, it has been focused on studying the response to therapy and highlighting precision medicine in studies of cancer immunotherapy, instead of characteristics of tumor or TME, due to limitations from target molecular layers (e.g., metabolome and lipidome) [107]. The proportion of metabolomic approaches for ICIs is inferior to that of genomics or transcriptomics. However, recent evidence indicated that microbiota as a source of metabolites had been involved in various diseases, including cancers and the immune system related to the tumor microenvironment [108]. Nevertheless, the identification of effects driving alterations to the immune system in tumors and in response to ICIs have not been fully understood yet due to the complexity of bacteria and its metabolites. Fortunately, advanced metabolomics techniques allow for high-throughput data acquisitions to understand this complexity. Given that cancer metabolism, especially the cancer immune system, is affected by microbiota directly or metabolites produced from bacteria indirectly, the identification of the microbiome and metabolome becomes a priority for omics research. Therefore, metabolomics combined with metagenomics are widely used to study the microbiome for cancer immunotherapy. Several previous studies have demonstrated that microbial metabolites and microbiota itself influence the efficacy of immunotherapy [109,110]. Although other omics approaches have contributed to suggest various roles of the microbiome in modulating the response to ICIs, comprehensive interpretation, including metagenomics and metabolomics, have been widely utilized to discover unknown interactions between the microbiome and ICIs [111,112]. Through this approach, specific bacterial strains or mechanisms for enhancing the efficacy of ICIs have been studied. For example, the specific gut microbiome of nivolumab and pembrolizumab responders was identified, while the synergistic effect of anti-PD-1 and *B. bifidum* strains reportedly reduced cancer growth by modulating the production of IFN- γ by intensifying biosynthesis of immune-stimulating metabolites [102,113]. The development of techniques and information regarding how the microbiome interacts with ICIs have promoted the discovery of microbiota-linked biomarkers for response prediction of ICIs (e.g., indole, aldehydes, and short-chain fatty acids), which could be a promising target for precision medicine [99].

### 3.6. Omics Approaches for Investigating Upstream Molecular Levels of the Metabolome

In systems biological approaches for cancer immunotherapy, especially for ICIs, proteogenomics and transcriptomics are frequently used because aberrant expression of neoantigens, microsatellite instability (MSI), DNA repair, and TMB are closely associated with abnormal effects of molecules from genome to proteome in the immune system. For example, Anagnostou et al. studied the initial response of ICIs from the comparison of pre-treatment and post-treatment, thus observing an association between genomic alteration and loss of mutation-associated neoantigens in resistant tumors, which demonstrated decreased therapeutic benefits [114]. Additionally, proteomics studies involving the secretome derived from T-cells and B-cells demonstrated specific protein signatures in the exosomes of patients receiving PD-1 inhibitors before treatment and in tumor-associated B-cells, suggesting that these protein signatures can be used as promising predictive markers for PD-1 inhibitors regarding activation of PD-1^+^ T-cells treated with PD-1 inhibitors [115,116]. Usually, genomics, transcriptomics, and proteomics are simultaneously applied and integrated to obtain synergetic interpretation beyond individual explanations. A previous study generated cell-type immune enrichment scores based on proteogenomic approaches, providing gene and protein expression levels of targets containing PD-1 and evaluating different types of glioblastoma [117]. Further, noninvasive identification methods to assess response at the early stage of ICI treatment were recommended using pre-treatment circulating tumor DNA and peripheral CD8+ T-cell levels to predict the durable clinical benefit of patients based on whole-exome sequencing and RNA-sequencing in non-small cell lung cancer [118]. Meanwhile, several studies reported the key roles of epigenetic markers in oncogenesis and immune-editing [119,120]. Recently, the EPIMMUNE signature was introduced by Duruisseaux et al., which encompassed specific patterns of DNA methylation from nivolumab- or pembrolizumab-treated non-small-cell lung cancer patients and was associated with clinical benefit [121].

Several recent studies using genomics and transcriptomics demonstrated that high MSI and TMB correlate with tumor antigenicity and the response to immunotherapy [122,123]. Evrard et al. mentioned that deficient DNA mismatch repair (dMMR) and MSI display heterogeneity originating from testing methods, and dMMR/MSI screening may be useful with TMB, regarding benefits from immunotherapy in colorectal cancers [124]. Meanwhile, Vanderwalde et al. performed MSI assays using NGS methods to highlight the relationship between MSI, TMB, and PD-L1 using over 11,000 patients across cancer types and suggested MSI as a marker with TMB and PD-L1 expression to determine the use of ICIs [125]. Although previous studies were not able to provide standardized TMB cutoffs among each study and cancer types, TMB is strongly considered as an independent predictive biomarker for the response to ICIs, while genomic techniques for reproducible TMB calculations are in continuous development [122].

## 4. Multidisciplinary Approaches beyond Fundamental Omics Studies for ICIs

Studies involving ICIs have been performed not only with individual omics but also with various other approaches. In this section, we will demonstrate how other approaches combined with omics have contributed to more comprehensive insights into cancer immunotherapy.

### 4.1. Multi-Omics and Multi-Layer Omics

Systems biology is an approach to understand the biological system at a diverse level (e.g., genes, proteins, and metabolites) [126]. Systems biology provides a powerful premise and promise based on multiple omics approaches generating high-throughput data sets for biological interpretation. Over the past decades, the development of technologies and reduction of costs facilitate the application of omics. These trends have motivated the use of multi-omics to improve the biological insight of research. Multi-omics or pan-omics is a comprehensive omics approach integrating the data sets from multiple omics (e.g., genomics, transcriptomics, proteomics, and metabolomics) to explain interactions among omics dimensions [127]. Recently, the ‘Australian and New Zealand Metabolomics Conference’ (ANZMET 2018) hosted a peer session on multi-omics, discussed potential limitations of multi-omics, and recommended strategies to overcome these limitations. Furthermore, several strategies to interpret biological meanings, such as top-down and bottom-up data reduction integration, or post-analysis data integration approaches and integrated data analysis approaches, were introduced [63]. Among strategies, post-analysis data integration is widely used and includes the key features on different omics dimensions. In contrast with data integration after analysis, integrated data analysis approaches merge various dimensions of omics data through specialized tools, and several studies have developed the methods and tools using different aspects [63,128].

To obtain valuable insight from massive omics information and complexity of the disease, the optimal integration methods are situationally applied, such as pathway data integration, network analysis, and statistical integration [64]. In oncology, the number of studies using multi-omics has been continuously increased for drug discovery, biomarker signature establishment for prognosis or diagnosis, and assessment of drug response. For example, Lindskrog et al. demonstrated a framework for biomarker discovery based on transcriptomics classification of non-muscle-invasive bladder cancer through a combination of transcriptomics and proteomics [129]. Several data repositories (e.g., TCGA, CPTAC, and METABRIC) about cancer established and have been frequently utilized in multi-omics [62]. Vasaikar et al. also tried to establish the LinkedOmics database containing clinical data derived by multi-omics techniques for 23 cancer types and data from TCGA to support multi-omics platforms for future studies [130]. Additionally, these multi-omics studies have promoted the emergence of precision medicine, considering individual or group variability for disease treatment and prevention [131].

A convenient way to perform comprehensive omics interpretation is the application of established omics databases. Project HOPE (High-tech Omics-based Patient Evaluation) is a clinical research project by the Institutional Review Board of Shizuoka Cancer Center in Japan, including comprehensive whole-exome sequencing and gene expression profiling for 1000 tumor tissues. Using HOPE, Akiyama et al. demonstrated the availability of a multi-omics database for investigating ICI targets, in that this approach provided seven immune response-associated genes and discovered over-expression of PD-L1 in hypermutators [132]. In addition, 13 melanoma data, including two responders from five nivolumab-treated patients in HOPE, were used to suggest upregulation of PD-L1 protein and increase single nucleotide variants after complete remission [133]. While using established omics databases, often the data is integrated with results from additional experiments to expand dataset types. For example, the relationship between VLCFA-containing lipids with ICI response via upregulation of peroxisome signaling in T-cells was indicated based on the integration of transcriptome data in TCGA with a metabolomics dataset from urological cancer patients [95]. In addition, comprehensive interpretation by nuclear magnetic resonance-based metabolomics, proteomic data, and TCGA provided choline kinase-α, cylcooxygenase-2, and transforming growth factor-β as promising options for combinatorial therapies based on ICIs [91].

Established databases can be used by extracting novel results and integrating more than two datasets through statistics or specialized tools for meta-analysis. Integration of TCGA and Gene Expression Omnibus (GEO) datasets involving head and neck cancer concluded that EGFR and PTGS2 were identified as important nodes of the immune phenotype-related network in genetic and epigenetic levels, and EGFR inhibition was recommended as a potential target of combination therapy for ICI non-responders [134]. Similarly, unsupervised clustering of data from 1000 patients with hepatocellular carcinoma from GEO, TCGA-LIHC, and ICGC classified three clusters and revealed potential predictive signatures for response and prognosis assessment of anti-PD-1 and anti-CTLA-4 immunotherapies [135]. Moreover, a previous study established a tool to estimate ICI response predictors using previous large-scale omics data, published ICI trials, non-immunotherapy tumor profiles, and CRISPR screening on the web [136].

Without database utilization, various studies have applied multi-omics approaches to investigate ICIs, including mechanisms of actions, response prediction, and target discovery for combination therapy. Among diverse combinations of omics data, integration of genomics and transcriptomics is widely used. Genomic and transcriptomic features were used as test and validation sets, analyzed separately to represent genomic and transcriptomic characteristics, or integrated before analysis [137,138,139]. Furthermore, transcriptomics data is usually combined with a proteomics approach to demonstrate phenotype differences by transcriptome-affected protein alteration [129]. However, few studies focus on ICIs based on multi-omics, including metabolomics, indicating that the expression of tumor- or immune-related proteins and mechanisms of action are the most effective and important targets for cancer immunotherapy [117].

### 4.2. Single-Cell Omics

Given that the functions of organs are derived from comprehensive activities of organized individual cells, identifying changes in cells caused by disease is important. Single-cell omics techniques typically analyze specific molecular layers from the identical individual cell and provide cellular heterogeneity, enabling a more profound understanding of key biological mechanisms [140]. Recently, single-cell omics have developed continuously, and some protocols, such as single-cell RNA sequencing, are standardized and widely used [141]. In oncology, the complex interaction between tumor cells and the surrounding microenvironment hampers precise investigation of cellular functions for cancer growth and progression using omics based on bulk analysis. However, single-cell omics can demonstrate functional differences in cellular states within a tumor, which is associated with phenotypic differences driven by particular molecular layers. The usefulness of single-omics techniques, especially single-cell RNA sequencing, for future studies, has been additionally improved by the Human Cell Atlas, established by an international consortium, providing high-throughput data for classification and identification of cells, which can be potentially used for data-driven interpretation of specific diseases [142]. Although current single-cell techniques contain limitations, such as spatial information, scalability, and PCR errors, recent developments (e.g., spatial sequencing and comprehensive joint profiling technology) promise to overcome these limitations [143,144].

In cancer immunology, the TME is considered a complicated mixture of diverse cells (e.g., tumor cells, stromal cells, and immune cells) and non-cellular components (e.g., signaling molecules and extracellular matrix), which play key roles for anti-tumor immunity. To date, many studies have applied single-cell omics, mainly single-cell transcriptomics, to investigate heterogeneity and complicated cross-cellular interactions in the TME [145]. In addition, various trials have been performed to evaluate ICI treatment. Bassez et al. analyzed clinical samples from breast cancer patients who received only anti-PD1 or neoadjuvant chemotherapy before anti-PD1 using single-cell transcriptomics combined with proteome profiling to understand a subset of tumors responding to ICI. This study identified the association of T-cell expansion after anti-PD1 treatment with immunophenotypes and gene sets positively (e.g., expression of PRF1, GZMB, and CXCL13) or negatively (e.g., TCF7+, GZMK+ T-cells, and CX3CR1+, C3+ inhibitory macrophages) [146]. Sade-Feldman et al. applied single-cell RNA sequencing to profile transcriptomes of more than 16,000 individual immune cells derived from 48 patients with melanoma in a discovery set. Through the validation set, including ex vivo and in vivo studies, they demonstrated TCF7+ CD8+ T-cells as a predictive marker for positive clinical outcomes [147]. These studies suggested not only a strategy for discovering predictors and mechanisms of ICI action but also novel targets for the development of cancer immunotherapies.

## 5. Expanding the Knowledge of Systems Biology Studies for Cancer Immunotherapy

### 5.1. Evaluation of Immune-Related Adverse Events

Cancer immunotherapies have received attention as a novel generation of cancer treatment. However, unexpected immune-related adverse events (irAE) have been observed following increased use and clinical trials. A network meta-analysis using 36 phase 2 and 3 randomized trials reported that the range of probability is 54% to 76%, and the pooled incidence is 66.4% to 86.8% for all adverse events of five ICIs (atezolizumab, nivolumab, pembrolizumab, ipilimumab, and tremelimumab) [148]. The onset of irAEs has been reported at eight days to more than one year after initial trials of ICI treatment in any organ system [149]. ICIs induce irAEs through various mechanisms, including T-cell activation, cross-reactivity of immune cells and healthy cells, B-cell-mediated autoantibodies, and monoclonal antibody-mediated direct injury [150]. Several guidelines have been recommended for the therapeutic management of irAEs, with steroidal treatment being suggested as a prominent way for irAEs, excluding endocrine irAEs [151,152].

The prevention of irAEs is as important as therapeutic management. To prevent occurrences of irAEs before cancer immunotherapy, several studies suggested diverse biomarkers for response prediction and to determine the mechanisms of irAE as a potential target for prevention. A multi-omics approach demonstrated that lymphocyte cytosolic protein 1 and adenosine diphosphate dependent glucokinase might serve as biomarkers for irAE prediction by evaluating the association between omics data and irAE reporting odds ratios [153]. Meanwhile, Grigoriou et al. focused on transcriptomic reprogramming of regulator T-cells in blood and suggested inflammatory Treg reprogramming as an indicator for irAE development [154]. Another study concentrated on changes observed in B-cell expression after ICI treatment and indicated that early changes in B-cells after treatment may become a marker for risk of irAE in melanoma, based on single-cell RNA sequencing [155].

### 5.2. Beyond ICIs

In recent decades, the success of ICI-based cancer immunotherapy verified immune system control, resulting in anti-cancer effects and the promotion of further studies identifying novel targets or establishing various methods for immune-oncology treatment. Although ICIs are one of the most well-established methods of immunotherapy, other methods, including immune cell therapy, anti-cancer vaccines, and antibody-drug conjugates, have been developed to overcome the clinical limitation of ICIs [156]. Following this trend, the systems biology approach using omics platforms has been widely used in the development of these methods, as well as those involving ICIs.

The aim of the anti-cancer vaccine is the induction of T-cell responses, usually against specific antigens from tumors [157]. Cancer vaccines combined with ICIs or other immunotherapy may induce their maximized effects [158]. To identify effective neoantigens, Matsushita et al. and Robbins et al. performed whole-exosome sequencing, which identified a mutant marker of sarcoma and a potential correlation between mutated antigens from autologous tumor cells and clinical response in melanoma [159,160]. Unlike the method using anti-cancer vaccines, immune cell therapy using T-cell receptors (TCR), chimeric antigen receptors (CAR), and tumor-infiltrating lymphocytes (TIL) is the treatment of isolated immune cells from patients or genetically engineered immune cells to patients for activating their immune systems. Integrated analysis of proteomic and transcriptomic data sets by a specific algorithm involving acute myeloid leukemia (AML) was conducted by Perna et al. and suggested the concepts of generalizable combinatorial targeting strategies to uncover candidate targets for AML and other cancer studies [161]. High-throughput techniques are also used to enhance therapeutic efficacy and aid the discovery of novel targets. Lu et al. developed novel methods, including single-cell RNA sequencing and co-culture techniques for tumor-infiltrating lymphocytes and autologous antigen-presenting cells to increase the efficacy of adoptive T-cell therapy [162].

## 6. Concluding Remarks

To date, numerous metabolomics- and other omics-based studies for ICIs have been applied for the development of novel biomarkers, the evaluation or prediction of outcomes, and the identification of mechanisms of action. Furthermore, advanced analytical high-throughput techniques have been developed and optimized for various ICI studies. These studies may provide valuable information for future studies involving various cancer immunotherapy options as well as ICIs.

## Figures and Tables

**Table 1:** FDA approvals of ICIs from January 2011 to May 2021.

Drug Name	Active Ingredient	Approval Date	Mechanism of Action	Company	First Approved Indications
Yervoy	Ipilimumab	25 March 2011	CTLA-4-blocker	Bristol-Myers Squibb	Late-Stage Melanoma
Keytruda	Pembrolizumab	4 September 2014	PD-1 blocker	Merck & Co., Inc.	Advanced or unresectable melanoma
Opdivo	Nivolumab	22 December 2014	PD-1 blocker	Bristol-Myers Squibb	Unresectable or metastatic melanoma
Tecentriq	Atezolizumab	18 May 2016	PD-L1 blocker	Genentech Inc.	Urothelial carcinoma, the most common type of bladder cancer
Bavencio	Avelumab	23 March 2017	PD-L1 blocker	Emd Serono Inc.	Metastatic Merkel cell carcinoma
Imfinzi	Durvalumab	1 May 2017	PD-L1 blocker	Astrazeneca	Locally advanced or metastatic urothelial carcinoma
Libtayo	Cemiplimab-rwlc	28 September 2017	PD-1 blocker	Regeneron Pharmaceuticals	Cutaneous squamous cell carcinoma
Jemperli	Dostarlimab-gxly	22 April 2021	PD-1 blocker	GlaxoSmithKline	Endometrial cancer

CTLA-4, cytotoxic T-lymphocyte-associated protein 4; PD-1, Programmed cell death protein 1; PD-L1, Programmed death-ligand 1.

**Table 2:** Potential immune checkpoint inhibitors under clinical trials enrolled in ClinicalTrials.gov. The search conditions were as follows: status, recruiting and not yet recruiting studies; condition of disease, cancer; other terms, PD-1 or PD-L1 or CTLA-4 or checkpoint.

	ClinicalTrials.gov Identifier	Purpose	Study Population	Interventions	Status	Phase
1	NCT02694822	Evaluation	Advanced solid cancers and Advanced solid cancers refractory to PD-1	Drug: AGEN1884	Active, not recruiting	Phase1/2
2	NCT03989362	Combination	Cancer	Drug: Vopratelimab and Ipilimumab	Active, not recruiting	Phase 2
3	NCT03515629	Combination	NSCLC	Drug: REGN2810/Ipilimumab, REGN2810/chemo/Ipilimumab, and Pembrolizumab	Active, not recruiting	Phase 3
4	NCT04172454	Evaluation	Advanced Solid Tumors Melanoma	Drug: AK104	Not yet recruiting	Phase 1B/2
5	NCT03527251	Combination	NSCLC	Drug: Ipilimumab, SHR-1210	Unknown	Phase 1
6	NCT04326257	Combination	HNSCC	Drug: Nivolumab/Relatlimab and Nivolumab/Ipilimumab	Recruiting	Phase 2
7	NCT04868708	Combination	Recurrent or Metastatic Cervical Cancer	Biological: AK104 and Bevacizumab, Drug: Paclitaxel and Cisplatin or Carboplatin	Not yet recruiting	Phase 2
8	NCT03430063	Combination	Advanced NSCLS	Drug: SDREGN2810, SDREGN2810/Ipilimumab, and HDREGN2810	Active, not recruiting	Phase 2
9	NCT04140526	Combination	NSCLC/Advanced Solid Tumor, Metastatic Melanoma, Metastatic Head and Neck Carcinoma, Metastatic RCC/Metastatic CRC, Sarcomas/Metastatic Prostate Cancer, Ovarian Cancer/SCLC, Metastatic Breast Cancer	Drug: ONC-392 and Pembrolizumab	Recruiting	Phase 1
10	NCT04544644	Combination	NSCLC	Drug: AK104/Anlotinib	Not yet recruiting	Phase 2
11	NCT02403193	Evaluation and Combination	NSCLC	Drug: PBF-509_(80~640 mg), PBF-509 (160~640 mg) + PDR001, RP2D with ICIs naïve, Experimental: RP2D with ICIs treated	Active, not recruiting	Phase1/2b
12	NCT02535078	Combination	Malignant Melanoma	Drug: IMCgp100, Durvalumab, Tremelimumab	Active, not recruiting	Phase 1B/2
13	NCT03388632	Combination	Metastatic Solid Tumors and Treatment-Refractory Cancers	Drug: rhIL-15, Ipilimumab, and Nivolumab	Recruiting	Phase 1
14	NCT03608046	Combination	Colorectal Neoplasms, Malignant	Drug: Avelumab, Cetuximab Injection, Irinotecan	Recruiting	Phase 2
15	NCT03040791	Expansion	Prostate Cancer	Drug: Nivolumab	Recruiting	Phase 2
16	NCT02821754	Combination	Biliary Tract Neoplasms, Liver Cancer, HCC, Cholangiocarcinoma, and Bile Duct Cancer	Drug: Durvalumab and Tremelimumab, Procedure: TACE, RFA, Cryoablation	Recruiting	Phase 2
17	NCT03019003	Combination	Head and Neck Cancer	Drug: Oral Decitabine and Durvalumab	Recruiting	Phase 1 and Phase 2
18	NCT03202758	Combination	Metastatic CRC	Drug: Durvalumab/Tremelimumab/FOLFOX	Unknown	Phase 1 and Phase 2
19	NCT02938793	Combination	Cancer	Drug: Durvalumab and Tremelimumab	Recruiting	Phase 2
20	NCT03925246	Expansion	High Grade Glioma/ Brain Cancer	Drug: Nivolumab	Active, not recruiting	Phase 2
21	NCT03084471	Combination	Advanced Solid Malignancies	Biological: MEDI4736 and MEDI4736/Tremelimumab	Active, not recruiting	Phase 3
22	NCT03608046	Combination	Colorectal Neoplasms, Malignant	Drug: Avelumab, Cetuximab Injection, and Irinotecan	Recruiting	Phase 2
23	NCT03409198	Combination	Breast Cancer, Hormone Receptor Positive Tumor, and Metastatic Breast Cancer	Drug: Ipilimumab, Nivolumab, Pegylated liposomal doxorubicin, and Cyclophosphamide	Active, not recruiting	Phase 2
24	NCT04319224	Combination	Cancer	Drug: Vopratelimab, Ipilimumab, Nivolumab	Recruiting	Phase 1 and Phase 2
25	NCT03526185	Combination	Metastatic Melanoma	Drug: Tumor Infiltrating Lymphocytes and Nivolumab/Ipilimumab	Active, not recruiting	Early Phase 1
26	NCT03911557	Combination	Tumor, Solid	Drug: Durvalumab/Tremelimumab	Recruiting	Phase 2
27	NCT03308396	Combination	Advanced Kidney Cancer, Kidney Cancer, and Clear Cell RCC	Drug: Guadecitabine, Durvalumab	Active, not recruiting	Phase 1 and Phase 2
28	NCT03186326	Expansion	Metastatic CRC MSI	Drug: FOLFOX regimen, FOLFIRI Protocol, Avelumab, Panitumumab, Cetuximab, Bevacizumab, and Aflibercept	Active, not recruiting	Phase 2
29	NCT03206073	Combination	CRC, Colorectal Carcinoma, Colorectal Adenocarcinoma, Refractory Cancer, and Colorectal Neoplasms	Drug: Durvalumab and Tremelimumab, Biological: Pexa-Vec	Active, not recruiting	Phase 1 and Phase 2
30	NCT03373760	Combination	Recurrent Squamous Cell Lung Carcinoma and Stage IV Squamous Cell Lung Carcinoma AJCC v7	Biological: Durvalumab and Tremelimumab, Other: Laboratory Biomarker Analysis	Active, not recruiting	Phase 2
31	NCT03959293	Combination	Gastric Adenocarcinoma and Gastric Cancer	Drug: Durvalumab, Tremelimumab, and FOLFIRI Protocol	Recruiting	Phase 2
32	NCT03693612	Combination	Neoplasms	Drug: Feladilimab, Tremelimumab, Docetaxel, Paclitaxel, and Cetuximab	Active, not recruiting	Phase 1 and Phase 2
33	NCT03755739	Administration	Hepatocarcinoma/Lung Cancer, Melanoma/Renal Cancer, Head and Neck Cancer, and Pancreas Cancer/Ovarian Cancer, CRC/Cervical Cancer/Breast Cancer	Drug: ICIs	Recruiting	Phase 2 and Phase 3
34	NCT03033576	Combination	Advanced Melanoma, Melanoma of Unknown Primary, Mucosal Melanoma, Refractory Melanoma, Stage III Cutaneous Melanoma AJCC v7, Stage IIIA Cutaneous Melanoma AJCC v7, Stage IIIB Cutaneous Melanoma AJCC v7, Stage IIIC Cutaneous Melanoma AJCC v7, Stage IV Cutaneous Melanoma AJCC v6 and v7, Unresectable Cutaneous Melanoma, and Unresectable Melanoma	Biological: Ipilimumab and Nivolumab	Active, not recruiting	Phase 2
35	NCT02821754	Combination	Biliary Tract Neoplasms, Liver Cancer/HCC, Cholangiocarcinoma, and Bile Duct Cancer	Drug: Durvalumab and Tremelimumab, Procedure: TACE, RFA, and Cryoablation	Recruiting	Phase 2

Administration, clinical trials for comparison of efficacy following different administration; AGEN1884, anti-CTLA-4 antibody; AK104, humanized IgG1 tetrameric PD-1/CTLA-4 bispecific antibody; chemo, chemotherapy; combination, clinical trials for novel drug combination regimen; CRC, colorectal cancer; evaluation, clinical trials for development of novel drug candidates; expansion, clinical trials for expansion of indication; FOLFOX, combination chemotherapy made up of folinic acid, fluorouracil, and oxaliplatin; HCC, hepatocellular carcinoma; HDREGN2810, high dose cemiplimab; HNSCC, head and neck squamous cell carcinoma; ICI, immune checkpoint inhibitor; IMCgp100, engineered T cell receptor specific for a peptide antigen derived from the protein gp100; MEDI4736, durvalumab; NSCLC, non-small cell lung cancer; ONC-392, a humanized anti-CTLA4 IgG1 monoclonal antibody; PBF-509, Adenosine A2a receptor antagonist; PDR001, anti-PD-1 antibody; pexa-Vec, a thymidine kinase gene-inactivated oncolytic vaccinia virus engineered for the expression of transgenes encoding human granulocyte-macrophage colony-stimulating factor (GM-CSF) and beta-galactosidase; RCC, renal cell carcinoma; REGN2810, cemiplimab; RFA, Radiofrequency ablation; rhIL-15, recombinant interleukin-15; RP2D, PBR-509 + PDR001; SCLC, small cell lung cancer; SDREGN2810, standard dose cemiplimab; SHR-1210, anti-PD-1 antibody; TACE, Transarterial chemoembolization.

**Table 3:** Metabolomics-based studies related to ICIs or focusing on the development of ICIs.

	Purpose	Related ICIs	Sample		Methods	Comments	Reference
			Subjects (Number)	Matrix			
1	Target discovery	-	in vitro	T-cells	LC-MS/MS	PD-1 signaling results in metabolic dysregulation, which suggests considerable metabolic interventions of ICIs’ efficacy.	[81]
2	Target discovery	-	in vitro	T-cells	LC-MS/MS	Mechanistic association between T-cell senescence and aberrant lipid metabolism was introduced as a novel target for cancer immunotherapy.	[82]
3	Target discovery	-	in vitro and ex vivo (11 patients with nivolumab and TIL therapy)	T-cells and TILs	LC-MS/MS	Sirt2, associated with reprogramming T-cell metabolism, was identified as a new target of cancer immunotherapy.	[83]
4	Target discovery	-	in vivo and patients with glioblastoma	tissue	LC-MS/MS and GC-MS	IDO1 inhibition mitigated radiation-induced immunosuppression in glioblastoma.	[84]
5	Target discovery and Biomarker suggestion	Nivolumab, Pembrolizumab	ICI-treated patients with NSCLC (23) vs. heathy subjects (20)	plasma	LC-MS/MS	IDO1 inhibitors are a promising treatment for NSCLC considering IDO1 activity seemed to a key role in the primary resistance of ICIs.	[85]
6	Target discovery	Anti-mouse PC-1, Nivolumab	in vitro, patients with glioblastoma (4), and patients with metastatic melanoma (4)	tissue	LC-MS/MS	ICIs induced the IL4I1, which facilitates tumor progression.	[86]
7	Target discovery	Anti-PD-1	in vivo and patients with HCC (196) vs. healthy subjects (176)	urine	LC-MS/MS	PRMT5 inhibition demonstrated a synergistic mechanism enhancing anti-tumor immunity and alleviated the resistance to ICIs.	[87]
8	Target discovery	Anti-mouse PD-1	in vivo	tissue	LC-MS/MS	nSMase2 overexpression increased anti-PD-1 efficacy in murine melanoma models.	[88]
9	Target discovery	-	patients with breast cancer (65)	tissue	MALDI-MSI	The accumulation of PI(18:0/20:3) may affect the PD-1-associated immune checkpoint pathway.	[89]
10	Target discovery	-	in vivo	plasma	LC-MS/MS and GC-MS	*KEAP1*/*NRF2* pathway alteration induced reprogramming of pentose phosphate pathway connected with tumorigenesis and tumor regression by immune checkpoint inhibition in NSCLC.	[90]
11	Target discovery	-	in vitro	Breast cancer cells and PDAC cells	^1^H-MRS	Chk-α, COX-2, and TGF-β mediated PD-L1 regulation of metabolism.	[91]
12	Target discovery	-	patients with breast cancer (58) and patients with HCC (29)	data from previous studies	-	UCD is related to an enhanced response to ICI therapy.	[92]
13	Target discovery and Biomarker suggestion	Anti-mouse PD-1 and Anti-mouse CTLA-4	in vitro and patients with PDAC	PDAC cells, serum, and tissue	NMR	IL17 inhibitor enhances ICI sensitivity, and tumor lactate was suggested as a promising early biomarker for efficacy of IL17/PD-1 combination.	[93]
14	Target discovery and Biomarker suggestion	Nivolumab	nivolumab-treated patients with advanced melanoma (78), nivolumab-treated patients with RCC (485), and everolimus-treated patients with RCC (349)	serum	LC-MS/MS	The combination of a PD-1 inhibitor with IDO/TDO inhibitors was suggested in that worse overall survival associated with simultaneous elevation of resistance and serum kynurenine/tryptophan ratio.	[94]
15	Biomarker suggestion	Nivolumab and Pembrolizumab	ICI-treated patients with urological cancer (28)	serum	LC-MS/MS	VLCFA-containing lipids are potential predictive biomarkers for ICIs’ response.	[95]
16	Efficacy evaluation	Nivolumab and Pembrolizumab	ICI-treated patients with NSCLC (19)	plasma	LC-MS/MS	Tryptophan metabolites may become potential predictive biomarkers for the efficacy of the ICIs.	[96]
17	Biomarker suggestion and Efficacy evaluation	Nivolumab and Pembrolizumab	ICI-treated patients with NSCLC (50)	serum	NMR	The metabolomic fingerprint of serum is a potential biomarker for the response of ICIs.	[97]
18	Method development	-	patients with melanoma (-)	stool	LC-MS/MS	A comprehensive approach to fecal sample collection and metabolites profiling of gut microbiome were demonstrated.	[98]
19	Biomarker suggestion	Nivolumab	nivolumab-treated patients with NSCLC (7), NSCLC patients without nivolumabtreatment (4) vs. healthy subjects (8)	stool	GC-MS/SPME and NMR	Microbiota-Linked Biomarkers, including SCFAs, were introduced through network analysis.	[99]
20	Efficacy evaluation	Nivolumab	nivolumab-treated patients with NSCLC (11)	stool	GC-MS/SPME and ^1^H-NMR	The identification of microbiota-linked “indicators” is a potential strategy for the prediction of responders, in that gut microbiota metabolic pathways affect the response of ICIs.	[100]
21	Biomarker suggestion	Nivolumab	nivolumab-treated patients with NSCLC (22)	serum and stool	GC-MS/SPME and NMR	An integrated parameter was proposed to identify good responders for nivolumab treatment.	[101]
22	Efficacy evaluation	Anti-mouse PD-1, Atezolizumab, Nivolumab, and Pembrolizumab	in vivo and ICI-treated patients with NSCLC (96) vs. healthy subjects (139)	serum and stool	LC-MS/MS	*Bifidobacterium bifidum* strains make a synergistic effect with ICIs to reduce tumor burden.	[102]
23	Efficacy evaluation	Nivolumab, and Pembrolizumab	ICI-treated patients of multiple cancers (52)	plasma and stool	LC-MS/MS	Fecal SCFA concentration may affect PD-1 inhibitors’ efficacy.	[103]
24	Efficacy evaluation	Nivolumab, Pembrolizumab, and Sintilimab	nivolumab-treated patients with NSCLC (4), pembrolizumab-treated patients with NSCLC (42), and sintilimab-treated patients with NSCLC (17)	stool	-	The correlation between intestinal microbiome β-diversity and the response of anti-PD-1 in NSCLC was indicated.	[104]

Chk-α, choline kinase-α; COX-2, prostaglandin-endoperoxide synthase 2; HCC, hepatocellular carcinoma; ICI, immune checkpoint inhibitor; IDO, indoleamine-2,3-dioxygenase 1; IL4I1, interleukin-4-induced-1; KEAP1, Kelch-like ECH-associated protein 1; KMO, kynurenine monooxygenase; KYNU, kynureninase; LC, liquid chromatography; MALDI, Matrix-Assisted Laser Desorption Ionization; MRS, magnetic resonance spectroscopy; MS/MS, tandem mass spectrometry; MSI, mass spectrometry imaging; NMR, nuclear magnetic resonance; NRF2, nuclear factor erythroid-2-related factor 2; NSCLC, non-small cell lung cancer; PBMC, peripheral blood mononuclear cells; PDAC, pancreatic ductal adenocarcinoma; PRMT5, Protein arginine N-methyltransferase 5; RCC, renal cell carcinoma; SCFA, short-chain fatty acid; Sirt2, NAD+-dependent deacetylase; TDO, tryptophan 2,3-dioxygenase; TGF-β, Transforming growth factor β; TIL, tumor-infiltrating lymphocytes; TN, triple-negative; UCD, urea cycle dysregulation; UV/Vis, UV-Vis spectrophotometer.

## Data Availability

Not applicable.

## Abbreviations

ACT	Adaptive cell transfer
ANOVA	Analysis of variance
APLCNR	Apelin receptor
C3	Complement component 3
CAR	Chimeric antigen receptor
CCL	Chemokine antigen receptor
CD137	Tumor necrosis factor receptor superfamily member 9 (TNFRSF9)
CD4+ cell	Cluster of differentiation 4-positive cell
CD8+ cell	Cluster of differentiation 8-positive cell
CRISPR	Clustered regularly interspaced short palindromic repeats
CTLA-4	Cytotoxic T-lymphocyte-associated protein 4
CX3CR1	CX3C chemokine receptor
CXCL13	Chemokine (C-X-C motif) ligand 13
cyTOF	Mass cytometry
dMMR	Deficient DNA mismatch repair
EGFR	Epidermal growth factor receptor
EI	Electron impact
ESI	Electrospray ionization
GC	Gas chromatography
GCMB	Glial Cells Missing Transcription Factor 2
GEO	Gene Expression Omnibus
GITR	Glucocorticoid-induced TNFR family-related gene
GZMK	Granzyme K
HOPE	High-tech Omics-based Patient Evaluation
IARC	International Agency for Research on Cancer
ICGC	International Cancer Genome Consortium
ICI	Immune checkpoint inhibitor
IDO	Indoleamine 2,3-dioxygenase
IFN	Interferon
IL	Interleukin
IL17RA	IL17 receptor A
irAE	Immune-related adverse event
LC	Liquid chromatography
LIHC	Liver hepatocellular carcinoma
MS	Mass spectrometry
MS/MS	Tandem mass spectrometry
MSI	Microsatellite instability
NGS	Next-generation sequencing
NIST	National Institute of Standards and Technology
NK	Natural killer cell
nSMase2	Sphingomyelinase 2
OX40	Tumor necrosis factor receptor superfamily, member 4 (TNFRSF4)
PCA	Principal component analysis
PCR	Polymerase chain reaction
PD-1	Programmed cell death protein 1
PD-L1	Programmed death-ligand 1
PEG	Polyethylene glycol
PLA2G4A	Phospholipase A_2_ group IVA
PLS-DA	Partial least squares-discriminant analysis
POLE	Polymerase epsilon
PRF1	Perforin 1
PTGS2	Prostaglandin-endoperoxide synthase 2
PTPN2	Protein Tyrosine Phosphatase Non-Receptor Type 2
TCF7	Transcription Factor 7
TCGA	The Cancer Genome Atlas
TCPA	The Cancer Proteome Atlas
TDO	Tryptophan 2,3-dioxygenase
TMB	Tumor mutation burden
TME	Tumor microenvironment
VEGFA	Vascular endothelial growth factor A
VISTA	v-domain Ig suppressor of T-cell activation
VLCFA	Very-long-chain fatty acid
VOC	Volatile organic compound
WGS	Whole-genome sequencing
WHO	World Health Organization
